# Importance of Molecular Data to Identify Fungal Plant Pathogens and Guidelines for Pathogenicity Testing Based on Koch’s Postulates

**DOI:** 10.3390/pathogens10091096

**Published:** 2021-08-28

**Authors:** Chitrabhanu S. Bhunjun, Alan J. L. Phillips, Ruvishika S. Jayawardena, Itthayakorn Promputtha, Kevin D. Hyde

**Affiliations:** 1 Innovative Institute for Plant Health, Zhongkai University of Agriculture and Engineering, Guangzhou 510225, China; avnishbhunjun@gmail.com; 2 Center of Excellence in Fungal Research, Mae Fah Luang University, Chiang Rai 57100, Thailand; ruvi.jaya@yahoo.com; 3 School of Science, Mae Fah Luang University, Chiang Rai 57100, Thailand; 4 Faculdade de Ciências, Biosystems and Integrative Sciences Institute (BioISI), Universidade de Lisboa, Campo Grande, 1749-016 Lisbon, Portugal; alan.jl.phillips@gmail.com; 5 Department of Biology, Faculty of Science, Chiang Mai University, Chiang Mai 50200, Thailand; itthayakorn.p@cmu.ac.th

**Keywords:** disease severity, image analysis, pathogenicity, phylogeny, plant disease assessment

## Abstract

Fungi are an essential component of any ecosystem, but they can also cause mild and severe plant diseases. Plant diseases are caused by a wide array of fungal groups that affect a diverse range of hosts with different tissue specificities. Fungi were previously named based only on morphology and, in many cases, host association, which has led to superfluous species names and synonyms. Morphology-based identification represents an important method for genus level identification and molecular data are important to accurately identify species. Accurate identification of fungal pathogens is vital as the scientific name links the knowledge concerning a species including the biology, host range, distribution, and potential risk of the pathogen, which are vital for effective control measures. Thus, in the modern era, a polyphasic approach is recommended when identifying fungal pathogens. It is also important to determine if the organism is capable of causing host damage, which usually relies on the application of Koch’s postulates for fungal plant pathogens. The importance and the challenges of applying Koch’s postulates are discussed. Bradford Hill criteria, which are generally used in establishing the cause of human disease, are briefly introduced. We provide guidelines for pathogenicity testing based on the implementation of modified Koch’s postulates incorporating biological gradient, consistency, and plausibility criteria from Bradford Hill. We provide a set of protocols for fungal pathogenicity testing along with a severity score guide, which takes into consideration the depth of lesions. The application of a standard protocol for fungal pathogenicity testing and disease assessment in plants will enable inter-studies comparison, thus improving accuracy. When introducing novel plant pathogenic fungal species without proving the taxon is the causal agent using Koch’s postulates, we advise the use of the term *associated with the* “*disease symptoms*” *of* “*the host plant*”. Where possible, details of disease symptoms should be clearly articulated.

## 1. Introduction

Animals and plants live in close contact with innumerable microorganisms, but only a small percentage can cause disease [1]. Fungi exhibit different types of associations with plants ranging from mutualism to parasitism [2]. They are vital in nutrient cycling which releases key plant nutrients into the soil [3]. As plant pathogens, they can cause significant damage in agriculture and forestry [4]. It was assumed that pathogenic microorganisms were fundamentally different from non-pathogenic microorganisms as they possess certain properties that are responsible for the pathogenic potential [5]. This led to the classification of microorganisms based on their pathogenic potential. This pathogen-centred view cannot be applied to certain organisms whose pathogenicity is dependent on the characteristics of the host rather than the microorganism [5]. *Candida albicans* and *Staphylococcus epidermitis*, for example, can cause diseases primarily in patients with impaired host defense or altered microbiota, whereas some microorganisms can be pathogens in normal individuals as well as those with impaired immunity [1]. Therefore, a pathogen cannot be defined based only on its ability to cause disease in hosts with impaired defense [5].

The definition of pathogen and host are intrinsically linked and host–pathogen interactions can be characterized based on whether they cause interruption of normal tissue structure or function of the host [1]. A pathogen can thus be described as an organism capable of causing host damage [6]. The host–pathogen interaction is one of the most complicated phenomena in biology [7]. Pathogens can be classified into biotrophs, necrotrophs, or hemibiotrophs based on their lifestyles [8]. Biotrophs such as rusts, powdery mildews, or *Peronospora* rely on living plant cells for nutrients [9]. Necrotrophs such as *Botrytis cinerea* or *Cochliobolus heterostrophus* rapidly kill the host tissues with toxins and cell wall degrading enzymes [9]. Hemibiotrophs such as *Pyricularia grisea* (*Magnaporthe grisea*), *Zymoseptoria tritici* (*Mycosphaerella* *graminicola*), and *Phytophthora infestans* have an initial period of biotrophy followed by the production of necrotrophic hyphae [9]. As a result, plants have evolved the ability to recognise and respond to pathogen, leading to rapid activation of defence responses [8].

Plant diseases can be caused by multiple fungal genera that affect diverse hosts with different tissue specificities involving a myriad of symptoms [7]. *Colletotrichum* species can cause anthracnose, foliar disease, rot, seedling blights, and post-bloom fruit drop in several hosts [10,11,12]. *Diaporthe* species are associated with root and fruit rots, dieback, stem cankers, leaf spots, and seed decay of a wide range of hosts [13]. *Pestalotiopsis* species can cause canker lesions, shoot dieback, leaf spots, needle blight, tip blight, grey blight, severe chlorosis, fruit rots, and post-harvest diseases in several economically important crops [4]. Fungal pathogens can cause devastating diseases that have a major financial impact and can also affect ornamental crops as well as the agricultural sector [10]. Some pathogens have led to the starvation of human populations such as *Phytophthora infestans*, which was responsible for the Irish potato famine, and *Bipolaris oryzae*, which was responsible for the 1943 Bengal famine in India [14]. It is estimated that fungal pathogens destroy one-third of all food crops annually [15]. Pathogenic fungi can also cause diseases in humans ranging from superficial infections to invasive infection that can lead to mortality [16]. It is estimated that fungal diseases are responsible for the death of over 1.6 million humans every year [16]. Therefore, a better understanding of pathogenicity, hosts, and methods of spread of fungal pathogens is vital for global biosecurity [10]. It is important to mention that diseases can also have positive impact in ecosystems. The “Tree Disease Concepts” discuss that forests need a “healthy amount of disease”, as these disease-causing agents act as regulators, terminators, and resource recovery agents, allowing the forest to be sustained over time [17]. This concept points out that forest health is only affected when the impact of pathogens exceeds a level suitable for the sustainability of the system.

In this review, we stress the importance of accurately identifying fungal pathogenic species using a polyphasic approach. If the fungus is plant-associated and a likely pathogen, this needs to be proven. We review the history, importance, and challenges of applying Koch’s postulates. We discuss limitations related to pathogenicity testing of plants pathogens. We provide guidelines for pathogenicity testing based on Koch’s postulates modified with aspects of Bradford Hill criteria incorporating the biological gradient criterion, the consistency criterion, and the plausibility criterion. A set of protocols is provided as a guideline for pathogenicity testing of fungal pathogens on different parts of the plant along with a severity score guide.

## 2. Species Identification Using Molecular Data

When identifying the cause of a plant disease, such as leaf lesions, it is important to accurately identify the fungal species, as well as confirm if the organism is capable of causing damage to the host [4]. This is because several organisms are present as endophytes or epiphytes on plant surface and do not cause disease; therefore, it is vital to confirm that the isolate is responsible for the pathogenicity [18]. Endophytes can become latent plant pathogens under certain inherent or environmental conditions or they can be conditional pathogens, which causes diseases as the plant ages or under stress conditions [19]. If the fungus is sporulating on the leaf lesion, it is relatively easy to identify the species to genus via its morphology [20,21,22,23,24], and obtain an isolate of the taxon via single spore isolation [25]. The isolate can then be used for pathogenicity testing and multi-gene molecular approaches for species-level identification [25]. If there is no sporulating fungus on the lesion, however, a different approach is needed. In this approach, the plant tissues are surface sterilized to minimize contaminants and the tissue is sectioned into small pieces that are placed on appropriate media, allowing for fungal growth [25]. This methodology is similar to the ones used for isolating endophytes [26,27], so it is not definitive that the causal agent will be isolated. This can result in the isolation of endophytes and pathogens, as this approach usually consists of the interphase or transitional area between healthy and infected tissue [18]. Therefore, it is important to inoculate the host with all the isolates to confirm that the correct isolate was obtained based on the observed symptoms. Sporulating isolates are identified to genus level via morphology and the isolates are identified to species level using multi-gene molecular approaches [25].

Pathogenic species were previously identified based only on morphology, which can lead to misidentification due to phenotypic plasticity [28]. Morphology-based identification was primarily based on spore characteristics [29]. This led to the implementation of different scientific names for species with different stages of sporing morphs, resulting in dual nomenclature [29]. Morphology-based identification resulted in recognizing morphospecies as a suite of indistinguishable taxa in several genera including *Phaeoacremonium*, which represent over 20 species based on molecular approaches [30]. Morphology-based identification is also a major limitation in several important pathogenic genera including *Bipolaris*, *Colletotrichum, Diaporthe*, and *Pythium*, as the species have overlapping morphology [10,31,32,33]. However, despite the limitations, morphology-based identification represents a fast way to identify isolates up to genus level [20,21,22,23,24].

Pathogenic species have also previously been identified based on their host association [34]. This resulted in morphologically similar fungi growing on different plant genera or plant species being given different scientific names [29]. The implementation of phylogenetic analyses provided a better understanding of species boundaries, which resulted in the implementation of using one name for one fungus [35]. Host-specificity alone cannot be used for species delineation, but it remains an important factor in biological control as a host-specific pathogen can have a limited distribution, whereas a taxon with a wide range of hosts is likely to be cosmopolitan [36]. However, our knowledge of host-specificity is limited as most studies have focused on economically important crops or ornamental crops [34]. A better understanding of host-specificity would require extensive sampling from different geographic locations and extensive examination of different parts of hosts using a combination of direct and high throughput methods [37]. 

Phylogenetic analyses have been used extensively to clarify species boundaries in several fungal genera [36,38,39]. However, there are several limitations associated with phylogenetic analyses for species identification [29]. Phylogenetic analyses do not account for hybridization events and horizontal gene transfer [29]. The internal transcribed spacer (ITS) region has been accepted as the universal barcode for fungi owing to the ease of amplification and its broad utility across the kingdom, but often, it can only be used for placement up to the genus level [40,41]. There are many species that have not been correctly identified in databases such as GenBank and there are also many unidentified species [31]. There is a lack of molecular data for several fungal species including reference sequences and some species only have ITS sequences, which hinders molecular-based approaches [31,33]. There is also a lack of ex-type or authenticated sequences for several pathogenic genera [36]. In some genera, there is no agreement as to the barcode that should be used for species-level identification [33]. Many pathogens have high genetic variation within individual species owing to the species’ historical dynamics, demography, and topography [42]. Pathogens have short generation times and large population sizes, which can lead to high levels of genetic variation owing to rapid adaptation to environmental factors and human-mediated factors [43]. The identification of species boundaries is thus important to better understand genetic variation in nature to develop sustainable control measures [43].

It is thus recommended to use a large taxon sampling based on multi-gene phylogeny of mitochondrial, nuclear, ribosomal, and protein-coding genes to accurately determine the phylogenetic relationships of taxa [29]. Novel taxa should ideally be introduced with more than one strain to provide insights into intraspecific phenotypic diversity and there should be reliable statistical support for the species relationship (at least 70% bootstrap or 0.90 posterior probabilities) [33]. It is also recommended to use different approaches including Bayesian inference, maximum likelihood, maximum parsimony coupled with automatic barcode gap discovery, coalescent-based methods, or the genealogical concordance phylogenetic species recognition to investigate species boundaries in pathogenic genera [31,33,36,44]. These approaches have been used to derive a better understanding of species boundaries in many important genera including *Alternaria*, *Bipolaris*, *Colletotrichum*, *Daldinia*, and *Diaporthe* [31,32,33,36,39,45,46]. Therefore, the application of a polyphasic approach based on morphology, ecology, and molecular-based approaches provides a solid framework for accurate species’ delineation [33,36,39].

### 2.1. Koch’s Postulates

Koch’s postulates are usually used to determine if an isolate is capable of causing host damage in plants [47]. Robert Koch began his work on disease transmission in 1873 when there was widespread interest in the control and prevention of several diseases [47]. He established novel techniques for the identification, isolation, and visualization of bacteria, which he used to identify and trace the life cycle of *Bacillus anthracis* [47]. He conducted animal inoculation experiments with anthrax bacilli to demonstrate that they caused anthrax [47]. In 1882, he discovered the microorganism Tuberkelvirus (*Mycobacterium tuberculosis*), which was responsible for pulmonary tuberculosis [48]. Koch then embarked on isolating the microorganism and he was awarded the Nobel Prize in Medicine in 1905 for his investigations and discoveries concerning tuberculosis [48]. Koch’s postulates were published in 1890 and they are often considered the first reliable method to establish whether a microorganism is the cause of disease [49]. These postulates were derived based on his work on infectious diseases such as anthrax and tuberculosis [49]. The postulates are commonly based on three basic concepts as follows: (a) the pathogen occurs in every occurrence of the disease; (b) the pathogen does not occur in other diseases as a fortuitous and non-pathogenic agent; and (c) after being fully isolated and repeatedly grown in pure culture, the pathogen can induce the disease again [50]. Koch concluded that, if all these conditions were met, then the occurrence of the pathogen in the disease could no longer be accidental [50]. The three concepts mentioned above are based on River’s translation [51]. In the absence of an animal model, Koch relied on naturally occurring processes that introduced the contagion, which was demonstrated by his findings on cholera based on outbreaks in villages [47].

### 2.2. Limitations of Koch’s Postulates

Koch recognized that his postulates had limitations as he stated that diseases such as anthrax, tetanus, and tuberculosis fulfilled all the postulates, while others did not [52]. Therefore, they do not apply to all pathogens. The first postulate is difficult to apply when the occurrence of the pathogen precedes the development of the symptoms [47]. This postulate cannot be applied to diseases whereby the toxins produced by the pathogen exert their effect at a site distant from the site of multiplication, such as in diphtheria [52]. The second postulate does not account for the presence of asymptomatic carriers and the third postulate cannot be applied to pathogens that cannot be cultured on artificial media [47]. Koch’s postulates often fail to accommodate the causal complexity characteristics of diseases [52]. Koch’s first two postulates can be classified as causal specificity, in which a given type of effect can have only one type of cause and a given type of cause can have only one type of effect [47]. This mono-causal model does not apply to all diseases, which can have multiple causes or risk factors, and one cause can have different effects [47].

### 2.3. Bradford Hill Criteria

The Bradford Hill criteria have been extensively used for inferring causation and have been used to evaluate countless hypothesized relationships between occupational and environmental exposures and disease outcomes [53]. In 1965, Bradford Hill published nine viewpoints for evaluating traditional epidemiologic data, but emphasised that they were neither necessary nor sufficient for causation [53]. The nine viewpoints include the strength of association, consistency, specificity, temporality, biological gradient, plausibility, coherence, experiment, and analogy [53]. Hill suggested that a strong association between exposure and disease is indicative of causation [54]. The consistency criterion is satisfied when there is a consistent association between two variables in different epidemiologic studies based on a variety of location, population, and methods [53]. The specificity criterion suggests that association is likely to be indicative of causation when they are specific, which means that the exposure causes only one disease [54]. The temporality criterion suggests that, for an exposure–disease relationship to be causal, the exposure must precede the onset of the disease [53]. According to the biological gradient criterion, the presence of a dose–response relationship supports the causal association between the exposure and the effect [55]. The plausibility criterion is satisfied if the relationship is consistent with the current knowledge regarding the aetiology and mechanism of disease [56]. The coherence criterion is satisfied if the cause-and-effect relationship follows the current knowledge [53]. The experiment criterion suggests that the strongest support for causal inference is a decline in disease risk following an intervention or cessation of exposure [56]. The analogy criterion is satisfied when there is strong evidence of a causal relationship between a particular agent and a specific disease, then weaker evidence could be accepted that a similar agent may cause a similar disease [53].

### 2.4. Limitations of Bradford Hill Criteria

The major limitation of the Bradford Hill criteria is that there is no method to decide whether to assign a checkmark and how to make a final assessment [53]. Along with the magnitude of the association between exposure and disease, the statistical significance is also important to determine the potential causality, which could be affected by the underlying method and other factors [56]. The limitation of the specificity criterion is the single-factor relationship of causal inference, but most diseases can have multiple causes or risk factors [53]. The specificity criterion cannot be applied for most infectious or complex diseases in which multiple pathogens can produce the same set of symptoms or a single pathogen can produce a number of outcomes [57]. The biological gradient criterion could also be seen as limited as most dose–response curves vary between studies owing to characteristics of the population, exposure routes, and individual susceptibility [58]. Several diseases occur as a result of the interplay and balance between multiple contributing and intermediary factors [53]. Therefore, it is difficult to demonstrate the biological plausibility of a causal relationship. Diseases result from multifaceted exposures that follow complex progression pathways and cessation of exposure may not reverse or slow the progression of the disease [56]. This limits the application of the experiment criterion. It has been argued that the modern application of the analogy criterion is not satisfied from confirming a causal inference, but from proposing and testing mechanistic hypotheses [53].

### 2.5. Limitations of Artificial Inoculations

It is important to artificially inoculate plants to determine if an isolate is capable of causing host damage [59]. Artificial inoculations are often conducted on detached or whole plants under extreme conditions that can favour the success of the infection [59]. The success of the infection often depends on the inoculum density, especially when using mycelium plugs, which provides an energy source for the pathogen [60]. The use of conidial suspension is recommended as the infection and spread of pathogens is likely to occur via conidia or ascospores [61]. Therefore, using conidial suspensions is more likely to produce a similar result as in nature, but mycelium plugs are often used when it is difficult to produce spore suspensions with enough conidia [61]. The process of surface sterilization is another factor that should be performed properly to remove surface microorganisms to ensure reliable results [26]. Pathogenicity tests conducted on detached plant tissues may not be reliable owing to suppression of the host defence pathways [62]. Artificial host inoculation is not reliable to determine host range, but the data can be used as an indicator of the infection potential [62]. Artificial inoculations are affected by environmental and physiological factors including temperature, humidity, and plant maturity [63]. Therefore, it is important to control abiotic factors such as temperature and humidity as they can influence the virulence of several pathogens during artificial host inoculation [1]. Fungicolous fungi are a diverse group of organisms that are associated with other fungi as symbionts, mycoparasites, or saprotrophs [64]. They can cause serious diseases of cultivated edible and medicinal mushrooms, but artificial inoculations cannot be performed for these fungicolous fungi to determine their pathogenic potential [64].

### 2.6. The Wounded versus Non-Wound Method

Plants are continuously exposed to environmental stress, which can cause wounding [65]. Wounding provides nutrients to pathogens and facilitates their entry into the plant tissues [65]. Artificial inoculations are performed using wounded and non-wounded methods and the wounded method should only be used for pathogens that normally enter through damaged plant parts [65]. These include most species of *Botryosphaeriaceae*, which can enter plant tissue via wounds [66].

### 2.7. Limitation When Dealing with Root and Stem Pathogens

To assess the severity of roots’ pathogens, the inoculated plants have to be carefully uprooted and examined at given sampling dates [67]. The severity of root pathogens can also be assessed based on features of the above-ground plant parts such as leaf mass and color [68]. The limitation of this method is that the relationship between the root disease and the features of the shoots has to be established [68]. Most studies dealing with root and stem pathogen are carried out using the wounded method, which favors the pathogen [69]. Artificial inoculation should be performed on hosts of similar age as the initial infection. This is a major limitation when dealing with stem and root pathogens of mature plants (>5 years) owing to the difficulty of acquiring hosts of similar age for pathogenicity testing. This sometimes results in species isolated from mature stems being tested for pathogenicity on young, tender shoots [70].

### 2.8. Virulence versus Aggressiveness Assessment

There are two kinds of pathogenicity, virulence and aggressiveness [71]. Virulence is a qualitative component and describes the ability of a genetically homogenous strain to grow on a genetically homogenous host [72]. When races of pathogen and varieties of the host interact differentially, the races are said to differ in virulence. When they do not interact differentially, they are said to differ in aggressiveness. Aggressiveness is a quantitative component of pathogenicity [72]. Aggressiveness is defined as the capacity of a natural pathogen to infect a host species (not possessing major resistance genes) or susceptible genotype [73]. These two concepts of pathogenicity must be kept separate and there is no known evidence for a positive correlation between virulence and aggressiveness [71]. 

### 2.9. Limitation of Studies When Introducing New Fungal Pathogens

Studies introducing new fungal pathogenic species usually include data on pathogenicity testing [13,74,75], but this has not been applied in all cases [76,77]. Inoculation of detached tissues allows disease assessment without destroying the whole plant, but it may not be reliable owing to the suppression of the host defence pathways [62]. Therefore, findings from detached inoculations should be regarded as preliminary findings that should be confirmed using the whole plant [62]. Another limitation of the detached method is difficulty in differentiating and describing the area of inoculation as the overall tissue changes colour after several days [74]. Different studies have used different scales in visual plant disease assessment, which is problematic for inter studies comparison. Some studies have used the nominal scale whereby the disease is graded as slight, moderate, or severe, but these scales have limited value as they are subjective and there is a lack of quantitative definition [68]. Some studies have used different tissues for pathogenicity testing to the one where the initial disease was observed [13]. Several studies have performed pathogenicity testing only in conditions of high humidity, which prevent the spores from drying out and enable germination, but it also favours the success of the infection [78]. There is also a lack of replication in some studies, which can lead to biased results [79]. There is a lack of cross pathogenicity testing in some studies, which is an important indicator of the infection potential [74].

### 2.10. Disease Assessment Based on Alternative Technologies

Disease assessment is important for the quantification of diseases and screening for resistance [68]. Inaccurate assessment of disease severity can lead to incorrect actions in disease management [68]. Visual assessment is one of the most widely used methods, but symptom severity assessed visually remains an estimate [80]. Owing to the limitations with visual assessments, several methods have been developed to detect and quantify diseases including image analysis and visible wavelength photography [79]. The main advantage of image analysis and visible wavelength photography is that they provide a non-destructive, non-invasive, and permanent record of the disease severity for future reference [79]. Image analysis has also been used to develop standard area diagrams that improve the accuracy and precision in visual assessment of disease severity [80]. Standard area diagrams have been developed for several important diseases including leaf spot of sunflower caused by *Alternaria*, rust of bean leaves caused by *Uromyces appendiculatus,* foliar disease in pyrethrum crops, and stem rust in seed crops of perennial ryegrass [81,82,83,84]. Standard area diagrams can also be generated using software by taking into consideration the leaf and disease symptoms, which allows its application to a range of diseases [80].

Visible wavelength photography has often been combined with image analysis to record, detect, or measure disease in plants [79]. It has also been used to study the host–pathogen interaction, especially disease resistance and pathogen aggressiveness [85,86]. The image of the diseased sample can be acquired using digital cameras or flatbed scanners and the image is edited using image processing software such as Adobe Photoshop (Adobe Systems, United States) [79]. Pixel colour is defined by the hue, intensity, and saturation, which are often used to separate healthy from diseased areas [87]. The number of pixels in the diseased versus healthy area is then used to calculate the percentage of the diseased area or lesion counts [88]. The differentiation of diseased versus healthy area can be a source of subjectivity, and it can also be difficult to distinguish between multiple diseases in the images [80]. The accuracy of measurements from image analysis can also be affected by several factors including focus, shadow, the reflection of light on the object, and uniformity of lighting [79].

The implementation of algorithms and statistical methods is important to estimate disease severity using image analysis [79]. Algorithms such as support vector machine can be used to reduce the incidence of false positives and false negatives, thus improving detection accuracy [89]. ASSESS is an important image analysis software in the field of plant pathology and it is aimed at measuring plant diseases [90]. This software has filters, contrast and colour saturation functions, as well as colour balancers to enhance the area of interest. It has high measurement accuracy and it also has an automated function [90]. There are several image analysis softwares that can differentiate between disease symptoms caused by different pathogens [91,92]. The software takes into account several criteria including lesion shape, lesion size, and texture, but this approach has only been successful in some cases [79]. Several statistical methods have been used to explore the quality of image analysis measurement [79]. Regression analysis has been widely used to assess accuracy, precision, reliability, and reproducibility in image analysis techniques [93,94]. The reliability or precision of the method is determined by the coefficient of determination [79]. Lin’s concordance correlation coefficient provides an unbiased and quantifiable method to investigate accuracy and precision in the image analysis technique [94,95]. Analysis of variance (ANOVA) and general linear modelling have also been used to investigate sources of error in disease severity estimation [96]. Another method is the coefficient of variation, which provides a good overall index as to the degree of precision and expresses the experimental error as a percentage of the mean [97].

## 3. Guidelines for Pathogenicity Testing in Plants

In this section, we provide guidelines for fungal pathogenicity testing, detailing the steps for inoculum preparation, host preparation, inoculation, and disease assessment. We provide guidelines for pathogenicity testing based on the application of a modified Koch’s postulate with Bradford Hill criteria to incorporate the biological gradient criterion (increased dose of inoculum leading to increased effect using suspension of different concentrations), the consistency criterion (similar outcome in different samples based on cross pathogenicity testing), and the plausibility criterion (the causal interpretation must not conflict with the current knowledge) [53].

### 3.1. Preparation of Inoculum

Pure cultures of the isolates are grown on suitable media at a suitable temperature [36]. An alternating 12 h fluorescent light and 12 h dark cycle can be used to induce sporulation [98]. Colonised mycelium plugs are obtained from the periphery of fungal colonies (0.5–1 cm from 14-day colonies) and, when inoculating, the mycelium plug is placed with the mycelium facing the plant tissue [69]. To harvest the conidia, 1–5 mL of sterilized distilled water is placed onto the culture, which is then gently swirled and scraped [36]. The conidial suspension is filtered through two layers of muslin to remove mycelium [36]. Conidial suspension of three different concentrations is prepared and the spore density is adjusted using a haemocytometer (10^4^ spores/mL; 1 × 10^6^ spores/mL; 2 × 10^6^ spores/mL). Conidial viability is evaluated by plating three aliquots (around 10 μL) of the suspension on suitable media and checking for further growth [99]. Only conidial suspensions with fungal growth are used for pathogenicity testing [99].

### 3.2. Host Preparation

All the experiments for the attached and detached methods should have replicates as mentioned below. A suitable part of the host is selected based on where the initial disease was observed [36]. It is recommended to use whole plants with at least three replicates (similar age as diseased host) for pathogenicity testing, but where unavailable, a suitable part of the host can be used in the detached method [36,74]. For the attached method, the suitable parts of the host are cleaned and surface sterilized as described below.

(a) Fruit and Leaf Preparation

For the detached method, it is recommended to have at least three replicates for fruits and leaves per treatment [74]. Freshly harvested, untreated, mature but unripe fruits (similar fruits as the initial infection), and leaves (similar size) are washed under running tap water for 1 min [36]. In the laminar flow cabinet, the fruits are surface-sterilized by washing in 70% ethanol for 3 min, then in 1% sodium hypochlorite for 3 min, followed by rinsing with sterilized distilled water three times [36]. The samples are surface dried with sterilized tissue paper and allowed to air-dry on sterilized filter paper [100].

(b) Petiole Preparation

For the detached method, petioles are cut to a uniform length (around 4 cm) [63]. The side leaflets are removed, leaving the petiole with only a central leaflet [101]. The petiole with the leaflet is surface-sterilized with 70% ethanol, then in 10% sodium hypochlorite for 10 min and allowed to air dry [63]. It is recommended to have at least 10 petioles per treatment [63].

(c) Cane and Stem Preparation

For the detached method, it is recommended to have at least 10 canes or stems per treatment (similar age and diameter as diseased host), which are cut to a uniform length (around 30 cm) [102]. All the leaves, prompt lateral branches, and tendrils are removed. They are surface-sterilized with 70% ethanol, then in 10% sodium hypochlorite for 10 min, and allowed to air dry [102]. This method can also be used for vascular pathogens. In the attached method for vascular pathogens, healthy potted plants with healthy roots are used [102]. The roots are washed to remove soil particles and transferred to pots containing sterilized soil. The shoot region to inoculate the vascular pathogen is surface sterilized with 70% ethanol, then in 10% sodium hypochlorite for 10 min, and allowed to air dry [102].

(d) Seed Preparation

The seeds are usually surface-sterilized by submerging in 0.1% Triton X-100 or 0.05% Silwet L-77 for 2 min, then in 0.5% sodium hypochlorite for 2 min, and 70% ethanol for 2 min (depending on the seeds) [103]. The seeds are rinsed three times in sterile distilled water and dried in sterilized paper towels [103]. Alternatively, the seeds can be surface-sterilized by submerging in 70% ethanol for 3 min, then in 2% sodium hypochlorite for 3 min, followed by rinsing with sterile distilled water three times and being allowed to air dry on sterilized filter papers in a laminar flow cabinet [104]. It is recommended to have at least 16 seeds per treatment [103].

The effectiveness of the surface sterilization method can be evaluated by plating aliquots of the final wash or by lightly pressing individual seeds on media plates and incubating the plates at room temperature for 10 days [105]. The disinfection is considered successful when no fungal growth is observed in the plate and seeds with fungal growth are discarded [105].

(e) Root Preparation

Pathogenicity testing for root pathogens depends on the type of the host and the time for the host to develop an adequate root system [69]. In the first method, seeds are surface sterilized as described and sown in sterilized soil [67]. After several weeks, the plants are carefully uprooted and the roots are washed to remove soil particles, surface sterilized with 1% sodium hypochlorite for 1 min, and washed thoroughly with sterile distilled water [106]. In the second method, seedlings (several years old) are acquired and only plants with asymptomatic roots are used for inoculation [69]. It is recommended to have at least five plants per treatment [69].

### 3.3. Inoculation

There are several inoculation methods for leaves and young shoots, which include spraying the spore suspension on the plant tissue [107], dipping the detached plant material into the spore suspension [99], or adding the spore suspension to the detached plant material [108]. The surface-sterilized fruits, leaves, or other parts of the plants are inoculated using the wound and non-wound technique [109].

For the attached method, buds, young shoots, and stems of woody plants can be inoculated by pricking with autoclaved map pins for the wound technique (16 mm needle length with ball-shaped top grip) [78]. A map pin is used to prick a hole in the buds, shoots, or stems and the same pin is used to pick up the inoculum, which is reinserted into the hole [78]. Sterile pins without inoculum are placed into control plants. The pins are left standing throughout the experiment until symptoms develop (usually 3–12 weeks) [78]. The different tissues can also be wounded using a sterilized blade and inoculated using conidial suspension or mycelium plugs as described below. For the control and non-wound technique, the different tissues are inoculated as described below. The inoculated plants are placed in a ventilated room at 25 °C [78]. All the experiments are repeated at a similar temperature and relative humidity to the collection site.

(a) Fruit and Leaf Inoculation

For the wound technique, the middle portion of the fruits and leaves are wounded using a sterilized blade [110]. The wounded fruits and leaves are inoculated by placing 6 μL of conidial suspension of different concentrations (10^4^ spores/mL; 1 × 10^6^ spores/mL; 2 × 10^6^ spores/mL) or mycelium plugs (2–5 mm) onto the wound [36]. The control fruits and leaves are inoculated with 6 μL of sterile distilled water or uncolonized plugs [36]. For the non-wound technique, 6 μL of conidial suspension of different concentrations is placed in the middle portion of the samples [98]. The infection site is covered with parafilm to maintain humidity at the start of the experiment, usually for 24–48 h (depending on the pathogen) [98]. All the inoculated samples are incubated individually in a moist chamber at 25 °C with a relative humidity of 80–90% for 7–14 days (or until symptoms develop, depending on the pathogen) [98].

(b) Petiole Inoculation

In the detached method, the petioles are placed in centrifuge tubes containing 0.5 mL mycelial conidial suspension of different concentrations [101]. The tubes are placed in a cryogenic storage box, which is then placed in a plastic box containing sterilized wet paper towels [101]. The top of the box is sealed using parafilm and the box is incubated in a greenhouse at 25 °C. This method can also be used to inoculate leaves [101].

(c) Cane and Stem Inoculation

Stem inoculation can be carried out by cutting open the bark and placing a conidial suspension of different concentrations or mycelium plug onto the wounded site [61]. The site is covered with parafilm to hold the inoculum [111]. Canes and stems can also be wounded using a 4 mm cork borer or a sterile disposable hypodermic needle [104]. The wounds are inoculated with 1 mL conidial suspension (different concentrations) or mycelium plugs. The wounds are covered with 100% pure Vaseline petroleum jelly and then wrapped with parafilm [111]. In the non-wounded methods, conidial suspension (different concentrations) or mycelium plugs are used to inoculate the samples, which are wrapped with parafilm [36]. The control canes and stems are inoculated with sterile distilled water or non-colonized plugs [36]. The inoculated canes and stems are then placed in transparent plastic containers containing sterilized wet paper towels to maintain a humid environment [111]. The inoculated samples are incubated at room temperature and inspected for lesion development [111]. For grapevine, the inoculated cuttings can be planted in individual pots containing sterilized soil or they can be placed in tubes containing sterile distilled water and placed in a greenhouse at 25 °C [61,112]. This detached method can also be used for vascular pathogens, whereby a 4 mm cork borer is used to wound the stem [111]. The wounded stems are inoculated using conidial suspension (different concentrations) or mycelium plugs. In the attached method, a wound is made (using a 4 mm cork borer) on the stem at around 10 cm above the soil level [69]. The wounds are inoculated with mycelium plugs of the vascular pathogen (uncolonized plugs for control) and covered with 100% pure Vaseline petroleum jelly, which are wrapped with parafilm [69].

(d) Seed Inoculation

Seed inoculation can be performed using dry conidia, soaking, or soil drenching [113,114,115]. The surface-sterilized seeds are soaked in conidial suspension of different concentrations containing 0.05% Silwet L-77 and, for the control, the seeds are soaked in sterile water [113]. The seeds are soaked in the dark at 25 °C for 24 h. The surface-sterilized seeds are planted in sterilized germination trays or plastic pots containing sterilized soil and are placed in a greenhouse at 25 °C under natural light to allow germination and growth [113]. The trays are watered when needed. In the second method, the surface-sterilized seeds are coated with the pathogenic strain by shaking them with dry conidia on a shaker at 80 rpm for 10 min before planting them [114]. In the third method, the seeds are first planted in germination trays or plastic pots containing sterilized soil. The soil in each germination tray or plastic pots is inoculated with 100 mL of the conidial suspension (different concentrations) containing 0.05% Silwet L-77, and the control is inoculated with 100 mL of sterile distilled water containing 0.05% Silwet L-77 [115]. They are then placed in a greenhouse at 25 °C under natural light and watered as needed [115].

(e) Root Inoculation

The surface-sterilized roots are inoculated by placing colonised mycelium plugs or conidial suspension of different concentrations on the roots and wrapping them with parafilm [106]. For the wound technique, the roots are wounded using a sterilized blade and inoculated by placing colonised mycelium plugs or conidial suspension (different concentrations) on the wound and wrapping them with parafilm [106]. For the control, sterile distilled water or uncolonized mycelium plugs are used. The inoculated samples are planted in pots containing sterilized soil or placed in tubes containing 40 mL Hoagland solution [106]. The inoculated samples are placed in a greenhouse at 25 °C under natural light and watered as needed [106].

### 3.4. Disease Assessment and Re-Isolation

Disease assessment is usually based on the evaluation of disease symptoms such as the appearance of lesion and the size of lesion according to their severity or incidence [116]. Disease severity is based on the percentage of the relevant host tissue or organ covered by symptoms, whereas disease incidence refers to the percentage of diseased plants or plant parts in the sample irrespective of their severity [68]. However, several factors can affect the estimate of severity including the size and shape of lesions as well as the colour and number of lesions [68]. Another important limitation is that the depth of the lesion is not taken into account in disease assessment [117,118].

We recommend including data from cross pathogenicity testing for all plant disease assessments. The disease reactions of the samples are evaluated from 7 to 14 days (up to several weeks depending on the tissue) after inoculation based on the disease symptoms [36]. The inoculated regions are cut longitudinally through the point of inoculation and the extent of necrosis is measured from the point of inoculation, excluding the wounded region from the measurements [102]. The area of the diseased tissue is calculated based on the length, width, and depth of the diseased symptoms. To evaluate the disease reaction of the root, the plants are carefully uprooted and the roots are washed to remove soil particles, surface sterilized using 2% sodium hypochlorite for 1 min, and washed thoroughly using sterile distilled water [67]. Root volume serves as a measure of the amount of root tissue lost due to rot [85].

Pathogenicity data are subjected to one-way analysis of variance [112]. It is recommended to assess disease severity using image analysis or visible-wavelength photography as the implementation of both image analysis and visual inspection will provide better accuracy and precision of severity measurement. Disease assessment is evaluated based on measurement of incidence and severity for visual inspection [116].

$$Disease\;Incidence=\left(\frac{Number\;of\;infected\;plant\;units}{Total\;number\;of\;plant\;units\;assessed}\right)\times 100\;Disease\;severity=\left(\frac{Area\;of\;diseased\;tissue}{Total\;tissue\;area}\right)\times 100$$

Aggressiveness is evaluated using the following disease severity scale for canes, fruits, leaves, petioles, seeds, and stems (Table 1 modified from [36]). We have modified the category and severity score from Cai et al. [36], which was only applicable to fruits.

Aggressiveness is evaluated using the following disease severity scale for roots (Table 2 modified from [106]). We have modified the category and severity score from Al-Sadi et al. [106], which included nominal scales for the disease severity, which can be subjective [80]. Table 2 includes five categories compared with eight categories in Table 1, as it includes three categories for disease severity under 10%. This is to allow the application of one scale accurately to individual plant organs (canes, fruits, leaves, petioles, seeds, and stems), which are of different sizes and are structurally different [80]. Only five categories are used to assess disease severity for roots, as most studies have used up to five severity categories and over 50% root with lesion are deemed as severe.

Koch’s third postulate is confirmed by re-isolating the inoculated fungus [112]. Pieces of the inoculated tissue are dissected from leading edges for re-isolation [67]. The identity of the re-isolated fungi is confirmed based on the disease symptoms as well as by sequencing the appropriate genes where possible. All inoculated samples are autoclaved twice before disposal [112].

## 4. Concluding Remarks

Fungal plant pathogens can cause serious host damage, but pathogenesis results from a complex interaction between the host immune system and the microorganisms that form part of the microbiota [1]. A polyphasic approach based on morphology, ecology, and molecular-based approaches is recommended to accurately identify pathogens as the scientific name links the knowledge concerning the species, which is vital to understand the epidemiology and to develop effective quarantine measures [31,36,39]. The Bradford Hill criteria and Koch’s postulates have several limitations, but they hold major importance in pathogenicity testing, especially for plant pathogens [47,53]. We provide guidelines for pathogenicity testing based on the application of an updated Koch’s postulate with Bradford Hill criteria to include the biological gradient criterion, the consistency criterion, and the plausibility criterion, whereby the causal interpretation must not conflict with the current knowledge [53]. We provide guidelines concerning testing different parts of the plant along with a guide for the severity score, which will lead to a uniform approach to pathogenicity testing and allow inter-studies comparison. The importance of using a suitable sample size to generate accurate mean and reliable results is reinforced [80]. We recommend assessing disease severity using visual inspection and image analysis as the implementation of both methods will provide better accuracy and precision of severity measurement. It is also important to use statistical analyses to investigate sources of error in disease severity estimation from both methods [112]. It is important to note that these are meant to guide causal inference and they should not be used as a heuristic for assessing causation, as there are no set rules that can account for all causations. Several pathogens do not fulfil all the requirements of Koch’s postulates as they cannot be cultured or they cannot produce the disease anew, but these pathogens are accepted as the cause of diseases with which they are associated [52]. Therefore, each criterion should be applied and interpreted based on each unique situation. We recommend all studies introducing novel plant pathogenic fungal species to include data on pathogenicity testing and we advise the use of the term *associated with the “disease symptoms” of “the host plant*” in studies that introduce novel species without proving the taxon is the causal agent using Koch’s postulates. Where possible, details of disease symptoms should be clearly articulated and a high-resolution image of the symptoms should be included for future reference.

## 5. Future Prospects

Several new methods are being developed to detect and confirm the pathogenic ability of microorganisms, especially those that currently cannot be cultured [119]. Multiplex PCR and real-time PCR have been used to amplify DNA regions coding for specific genes of the targeted pathogen [120]. A major limitation of most PCR techniques is that they cannot differentiate between viable and non-viable cells that led to the development of reverse transcriptase PCR, which is capable of differentiating viable cells [121]. Whole-genome sequencing technologies have also been used to study pathogens [122]. They have provided insights into genes that could be responsible for certain pathogenic traits, but the expression of these genes and the production of a diseased phenotype strongly depends on physiological and ecological factors [123]. Comparative genomics tools have been used in combination with several databases to assign putative functions to products of known or predicted fungal genes [119]. The identification of virulence factors is important to understand the complex processes involved in disease initiation, host immune activation, and the ability of the pathogen to cause an infection [119]. These approaches can be important to target pathogens from uncultured taxa detected by high throughput methods [119]. A major limitation for DNA-based approaches remains the absence of viable microorganism with which to reproduce the disease to satisfy Koch’s third postulate, but the insights from these genomic data are vital for the development of diagnostic tools for rapid and effective responses to disease outbreaks [124].

## Figures and Tables

**Table 1:** Disease severity scale for canes, fruits, leaves, petioles, seeds, and stems.

Category	Severity
1	no symptoms
2	1–2% of the sample with a necrotic lesion
3	>2 to 5% of the sample with a necrotic lesion
4	>5 to 10% of the sample showing a necrotic lesion
5	>10 to 25% of the sample covered with a necrotic lesion
6	>25 to 50% of the sample showing necrosis
7	>50 to 75% of the sample showing necrosis
8	>75% of the sample showing necrosis

**Table 2:** Disease severity scale for roots.

Category	Severity
1	no symptoms
2	1–10% of the root with a necrotic lesion
3	>11 to 25% of the root with a necrotic lesion
4	>25 to 50% of the root with a necrotic lesion
5	>50% of the root with a necrotic lesion

## Data Availability

Not applicable.
